# SeqWare Query Engine: storing and searching sequence data in the cloud

**DOI:** 10.1186/1471-2105-11-S12-S2

**Published:** 2010-12-21

**Authors:** Brian D O’Connor, Barry Merriman, Stanley F Nelson

**Affiliations:** 1 UNC Lineberger Comprehensive Cancer Center, University of North Carolina, Chapel Hill, NC, 27599, USA; 2 Department of Human Genetics, University of California, Los Angeles, CA, 90095, USA

## Abstract

**Background:**

Since the introduction of next-generation DNA sequencers the rapid increase in sequencer throughput, and associated drop in costs, has resulted in more than a dozen human genomes being resequenced over the last few years. These efforts are merely a prelude for a future in which genome resequencing will be commonplace for both biomedical research and clinical applications. The dramatic increase in sequencer output strains all facets of computational infrastructure, especially databases and query interfaces. The advent of cloud computing, and a variety of powerful tools designed to process petascale datasets, provide a compelling solution to these ever increasing demands.

**Results:**

In this work, we present the SeqWare Query Engine which has been created using modern cloud computing technologies and designed to support databasing information from thousands of genomes. Our backend implementation was built using the highly scalable, NoSQL HBase database from the Hadoop project. We also created a web-based frontend that provides both a programmatic and interactive query interface and integrates with widely used genome browsers and tools. Using the query engine, users can load and query variants (SNVs, indels, translocations, etc) with a rich level of annotations including coverage and functional consequences. As a proof of concept we loaded several whole genome datasets including the U87MG cell line. We also used a glioblastoma multiforme tumor/normal pair to both profile performance and provide an example of using the Hadoop MapReduce framework within the query engine. This software is open source and freely available from the SeqWare project (http://seqware.sourceforge.net).

**Conclusions:**

The SeqWare Query Engine provided an easy way to make the U87MG genome accessible to programmers and non-programmers alike. This enabled a faster and more open exploration of results, quicker tuning of parameters for heuristic variant calling filters, and a common data interface to simplify development of analytical tools. The range of data types supported, the ease of querying and integrating with existing tools, and the robust scalability of the underlying cloud-based technologies make SeqWare Query Engine a nature fit for storing and searching ever-growing genome sequence datasets.

## Background

Recent advances in sequencing technologies have led to a greatly reduced cost and increased throughput [1]. The dramatic reductions in both time and financial costs have shaped the experiments scientists are able to perform and have opened up the possibility of whole human genome resequencing becoming commonplace. Currently over a dozen human genomes have been completed, most using one of the short read, high-throughput technologies that are responsible for this growth in sequencing [2-16]. The datatypes produced by these projects are varied, but most report single nucleotide variants (SNVs), small insertions/deletions (indels, typically <10 bases), structural variants (SVs), and may include additional information such as haplotype phasing and novel sequence assemblies. Paired tumor/normal samples can additionally be used to identify somatic mutation events by filtering for those variants present in the tumor but not the normal.

Full genome sequencing, while increasingly common, is just one of many experimental designs that are currently used with this generation of sequencing platforms. Targeted resequencing, whole-exome sequencing, RNA sequencing (RNA-Seq), Chromatin Immunoprecipitation sequencing (ChIP-Seq), and bisulfite sequencing for methylation detection are examples of other important analysis types that require large scale databasing capabilities. Efforts such as the 1000 Genomes project (http://www.1000genomes.org), the Cancer Genome Atlas (TCGA, http://cancergenome.nih.gov), and the International Cancer Genome Consortium (http://www.icgc.org) are each generating a wide variety of such data across hundreds to thousands of samples. The diversity and number of sequencing datasets already produced, in production, or being planned present huge infrastructure challenges for the research community.

Primary data, if available, are typically huge, difficult to transfer over public networks, and cumbersome to analyze without significant local computational infrastructure. These include large compute clusters, extensive data storage facilities, dedicated system administrators, and bioinformaticians adept at low-level programming. Highly annotated datasets, such as finished variant calls, are more commonly available, particularly for human datasets. These present a more compact representation of the most salient information, but are typically only available as flat text files in a variety of quasi-standard file formats that require reformatting and processing. This effort is substantial, particularly as the number of datasets grow, and, as a result, is typically undertaken by a small number of researchers that have a personal stake in the data rather than being more widely and easily accessible. In many cases, essential source information has been eliminated for the sake of data reduction, making recalculation impossible. These challenges, in terms of file sizes, diverse formats, limited data retention, and computational requirements, can make writing generic analysis tools complex and difficult. Efforts such as the Variant Call Format (VCF) from the 1000 Genomes Project provide a standard to exchange variant data. But to facilitate the integration of multiple experimental types and increase tool reuse, a common mechanism to both store and query variant calls and other key information from sequencing experiments is highly desirable. Properly databasing this information enables both a common underlying data structure and a search interface to support powerful data mining of sequence-derived information.

To date most biological database projects have focused on the storage of heavily annotated model organism reference sequences. For example, efforts such as the UCSC genome databases [17], the Generic Model Organism Database**’**s Chado schema [18], and the Ensembl database [19] all solve the problem of storing reference genome annotations in a complete and comprehensive way. The focus for these databases is the proper representation of biological data types and genome annotations, but not storing many thousands of genomes worth of variants relative to a given reference. While many biological database schemas currently in wide use could support tens or even hundreds of genomes worth of variant calls, ultimately these systems are limited by the resources of a single database instance. Since they focus on relatively modest amounts of annotation storage, loading hundreds of genomes worth of multi-terabyte sequencing coverage information, for example, would likely overwhelm these traditional database approaches. Yet the appeal of databasing next generation sequence data is clear since it would simplify tool development and allow for useful queries across samples and projects.

In this work we introduce the SeqWare Query Engine, a scalable database system intended to represent the full range of data types common to whole genome and other experimental designs for next generation sequence data. HBase was chosen as the underlying backend because of its robust querying abilities using the Hadoop MapReduce environment and its auto-sharding of data across a commodity cluster based on the Hadoop HDFS distributed filesystem (http://hadoop.apache.org). We also present a web service that wraps the use of MapReduce to allow for sophisticated queries of the database through a simple web interface. The web service can be used interactively or programmatically and makes it possible to easily integrate with genome browsers, such as the UCSC Browser [20], GBrowse [21], or IGV (http://www.broadinstitute.org/igv), and with data analysis tools, such as the UCSC table browser [22], GALAXY [23], and others. The backend and web service can be used together to create databases containing varying levels of annotations, from raw variant calls and coverage to highly annotated and filtered SNV predictions. This flexibility allows the SeqWare Query Engine to scale from raw data analysis and algorithm tuning through highly annotated data dissemination and hosting. The design decision to move away from traditional relational databases in favor of the NoSQL-style of limited, but highly scalable, databases allowed us to support tens of genomes now and thousands of genomes in the future, limited only by the underlying cloud resources.

## Methods

### Design approach

**Figure 1:**
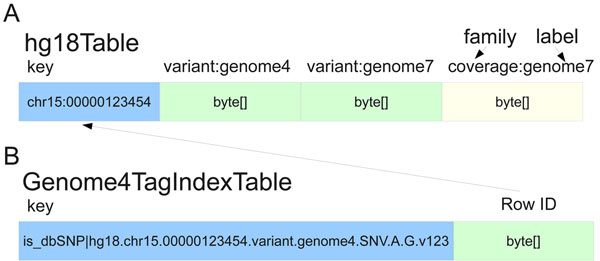
**SeqWare Query Engine schema**. The HBase database is a generic key-value, column oriented database that pairs well with the inherent sparse matrix nature of variant annotations. (a) The primary table stores multiple genomes worth of generic features, variants, coverages, and variant consequences using genomic location within a particular reference genome as the key. Each genome is represented by a particular column family label (such as “variant:genome7”). For locations with more than one called variant the HBase timestamp is used to distinguish each. (b) Secondary indexing is accomplished using a secondary table per genome indexed. The key is the tag being indexed plus the ID of the object of interest, the value is the row key for the original table. This makes lookup by secondary indexes, “tags” for example, possible without having to iterate over all contents of the primary table.

The HBase backend for SeqWare Query Engine is based on the increasingly popular design paradigm that focuses on scalability at the expense of full ACID compliance, relational database schemas, and the Structured Query Language (SQL, as reflected in the name “NoSQL”). The result is that, while scalable to thousands of compute nodes, the overall operations permitted on the database are limited. Each records consists of a key and the value, which consists of one or more “column families” that are fixed at table creation time. Each column family can have many “labels” which can be added at any time, and each of these labels can have one or more “timestamps” (versions). For the query engine database, the genomic start position of each feature was used as the key while four column families served to represent the core data types: variants (SNVs, indels, SVs, and translocations), coverage, features (any location-based annotations on the genome), and coding consequences which link back to the variants entries they report on. The coverage object stores individual base coverages in a hash and covers a user-defined range of bases to minimize storage requirements for this data type. New column families can be added to the database to support new data types beyond those described here. Additional column family labels are created as new genomes are loaded into the database, and timestamps are used to distinguish variants in the same genome at identical locations. Figure 1 shows an example row with two genomes**’** data loaded. This design was chosen because it meant identical variants in different genomes are stored within the same row, making comparisons between genomes extremely fast using MapReduce or similar simple, uniform operators (Figure 1a). The diagram also shows how secondary indexes are handled in the HBase backend (Figure 1b). Tags are a convenient mechanism to associate arbitrary key-value pairs with any variant object and support lookup for the object using the key (tag). When variants or other data types are written to the database, the persistence code identifies tags and adds them to a second table where the key is the tag plus variant ID and the value is the reference genomic table and location. This enables variants with certain tags to be identified without having to walk the entire contents of the main table.

### Datasets

Fourteen human genome datasets were chosen for loading into a common SeqWare Query Engine backend, see Table 1 for the complete list. Most datasets included just SNV, small indel, and a limited number of SV predictions. The U87MG human cancer cell line genome was used to test the load of large-scale, raw variant analysis data types. For this genome, SNVs, small indels, large deletions, translocation events, and base-by-base coverage were all loaded. For the SNV and small indels, any variant observed once or more in the underlying short read data were loaded, which resulted in large numbers of spurious variants (i.e. sequencing errors) being loaded in the database. This was done purposefully for two reasons: for this study, to facilitate stress testing the HBase backend, and for the U87MG sequencing project, to facilitate analysis algorithm development by giving practical access to the greatest potential universe of candidate variants. In particular, the fast querying abilities of the SeqWare Query Engine enabled rapid heuristic tuning of the variant calling pipeline parameters through many cycles of filtering and subsequent assessment.

**Table 1:** Datasets

Dataset	Technology	SNVs & Indels	SV	Translocations	Reference
European-Venter	Sanger	Y	Y	N	Levy *et al.* 2007 [3]
European-Watson	454	Y	Y	N	Wheeler *et al.* 2008 [4]
European- Quake	Helicos	Y	Y	N	Pushkarev *et al.* 2009 [5]
Asian	Illumina	Y	Y	N	Wang *et al.* 2008 [6]
Yoruban 18507	Illumina	Y	Y	N	Bentley *et al.* 2008 [7]
Yoruban 18507	SOLiD	Y	Y	N	McKernan *et al.* 2009 [8]
Korean	Illumina	Y	Y	N	Ahn *et al.* 2009 [9]
Korean-AKI	Illumina	Y	Y	N	Kim *et al.* 2009 [10]
3 human genomes	Complete Genomics	Y	Y	N	Drmanac *et al.* 2009 [11]
AML T/N	Illumina	Y	Y	N	Ley *et al.* 2008 [12]
AML genome	Illumina	Y	Y	N	Mardis *et al.* 2009 [13]
Melanoma	Illumina	Y	Y	N	Pleasance *et al.* 2010 [15]
Lung cancer	SOLiD	Y	Y	N	Pleasance *et al.* 2010 [14]
U87MG	SOLiD	Y	Y	Y	Clark *et al.* 2010 [16]

Fourteen whole genome datasets were loaded into the database, including the U87MG genome, with the March 2006 assembly of the human genome used as reference (NCBI36/hg18). Variant types (SNVs, small/large indels, SVs, etc) loaded and publication references are noted for each respective dataset. This table was adapted from Snyder *et al.* 2010.

A secondary dataset generated in our lab, the “1102 GBM” tumor/normal whole genome sequence pair, was used to compare the performance between the BerkeleyDB and HBase Query Engine backend types. This dataset, like the U87MG genome, included loading raw variant calls seen once or more in both backends in order to profile the load and query mechanisms.

### Programmatic access

The SeqWare Query Engine provides a common database store interface that supports both the BerkleyDB and HBase backend types. This store object provides generic methods to read and write the full range of data types into and out of the underlying database. It handles the persistence and retrieval of keys and objects to and from the database using a flexible object mapping layer. Simple to write bindings are created when new data types are added to the database. The underlying schema-less nature of key/value stores like BerkeleyDB and HBase make this process very straightforward. The store also supports queries that lookup all variants and filter by specific fields, such as coverage or variant call p-value, and it can also query based on secondary indexes, typically a tag lookup (key-value pair). The underlying implementation for each store type (BerkeleyDB or HBase) is quite different but the generic store interface masks the difference from the various import and export tools available in the project. The store interface was used whenever possible in order to maximize the portability of the query engine and to make it possible to switch backends in the future.

Two lower-level APIs are available for querying the HBase database directly. The first is the HBase API, which the store object uses for most of its operations including filtering variants by tags. This API is very similar to other database interfaces and lets the calling client iterate and filter result sets. HBase also support the use of a MapReduce source and sink, which allow for database traversal and load respectively. This was used to iterate over all variants in the database as quickly as possible and to perform basic analysis tasks such as variant type counting. The speed and flexibility of the MapReduce interface to HBase make it an attractive mechanism to implement future functionality.

### Web service access

The web service is built on top of the programmatic, generic store object and uses the Restlet Java API (http://www.restlet.org). This provides a RESTful [24] web service interface for the query methods available through the store. When loaded from a web browser, the web service uses XSLT and CSS to display a navigatable web interface with user-friendly web forms for searching the database. Queries on three data types are supported: variants, coding consequence reports, and per-base coverage. Variants can be searched by tag and also a wide variety of fields such as their depth of coverage. The tag field is used to store a variety of annotations and, in the U87MG database, this includes dbSNP status, mutational consequence predictions, and names of overlapping genes, among others. For the variant reports, the standard BED file type (http://genome.ucsc.edu/FAQ/FAQformat) is supported and users can alternatively select to load the query result directly in the UCSC browser, the IGV browser, or generate a list of non-redundant tags associated with the variant query results. Coverage information can be queried only by location, and the result can be generated in WIG format (http://genome.ucsc.edu/FAQ/FAQformat), WIG with coverage averaged by each block, or loadable links for the UCSC and IGV browsers.

When queried programmatically, the web service returns XML result documents. These contain enough metadata to construct URLs accessible from a wide variety of programming languages which can then be used to return query results in standardized formats (BED, WIG, etc). Since every query is just a URL, they can be created from within a web browser using the user-friendly form fields and cut-and-pasted into another tool or script. These URLs can then be shared over email, linked to in a publication, and bookmarked for later use, thereby providing a convenient, stateless, and universally interpretable reference to the results.

### Data load tools

Most of the genome datasets used in this project where limited to SNV, indel, and SV predictions. For those genomes, the variant information files that were available were loaded into the query engine using either standard file type loaders (GFF, key-value files, etc) or using custom annotation file parsers. The pileup file format (produced by SAMtools [25]) was used to load both the U87MG and “1102 GBM” tumor/normal genome variants and coverage information. The variant loading tool supports a plugin interface so new annotation file types can be easily supported. This import tool is available in the query engine package and uses the generic store interface for loading information in database backend (see “Programmatic Access” for more information). The HBase store currently uses an API very similar to other database connection APIs, and is therefore not inherently parallel, although multiple loads can occur simultaneously.

### Analysis tools

Two prototype analysis tools were created for use with the U87MG and “1102 GBM” tumor/normal databases. First, a MapReduce variant query tool was written to directly compare the performance of the retrieval of variants from HBase using the API versus using MapReduce. This simple tool used the HBase TableInputFormat object and a MapReduce job to traverse all database rows. The second analysis tool created was a simple somatic mutation detector for use with the “1102 GBM” tumor/normal genome database. Again, the HBase TableInputFormat object was used to iterate over the variants from the tumor genome, evaluating each variant by user-specified quality criteria in the map step and identifying those that were present in the cancer but not in the normal. In the reduce phase the coverage at each putative somatic mutation location was checked and only those where the coverage was good and no normal sample variant was called where reported as putative somatic mutations.

### Performance measurement

Backend performance was measured for both the BerkeleyDB and HBase stores using both data import and export times as metrics. The “1102 GBM” tumor/normal genome was used in this testing, which included enough variant calls (both true and spurious) to stress test both backends. A single threaded, API-based approach was taken for the load test with both the BerkeleyDB and HBase backends. Each chromosome was loaded in turn and the time taken to import these variants into the database was recorded. A similar approach was taken for the retrieval test except in this case the BerkeleyDB backend continued to use an API approach whereas the HBase backend used both an API and MapReduced approach. The export test was interleaved with the import test, wherein after a chromosome was loaded the variants where exported and both processes were timed. In that way we monitored both the import and export time as a function of overall database size.

The HBase tests were conducted on a 6 node HBase cluster where each node contained 8 2.4GHz Xeon CPUs, 24GB of RAM, and 6TB of hard drive space. Each node contained 16 map task slots and 4 reducer task slots. The BerkeleyDB tests were conducted on a single node, since it does not support server clustering, but with hardware identical to that used in the HBase tests.

## Results

### U87MG genome database

For our original U87MG human genome cell line sequencing project, we created the SeqWare Query Engine database, first built on BerkeleyDB (http://www.oracle.com/technetwork/database/berkeleydb) and then later ported to HBase (http://hbase.apache.org). For the work presented here, the U87MG database was enhanced with the addition of the 13 other human genome datasets that were publicly available when this effort commenced. To further enhance the query engine, we also added new query strategies and utilities, such as a MapReduce-based variant search tool. Unlike the U87MG genome, which included all variant calls regardless of quality, the other genomes included only post quality filtered variants. Still, they offer a proof of concept that the HBase backend can represent multiple genomes worth of sequence variants and associated annotation data. This sample query engine is hosted at http://genome.ucla.edu/U87 and can be used for both programmatic and interactive queries through the web service interface. A database snapshot is not available for download (due to its large size) but all the source datasets are publicly available and the database can be reconstituted in another location using either the BerkeleyDB or HBase backends along with the provided query engine load tools.

### Performance

**Figure 2:**
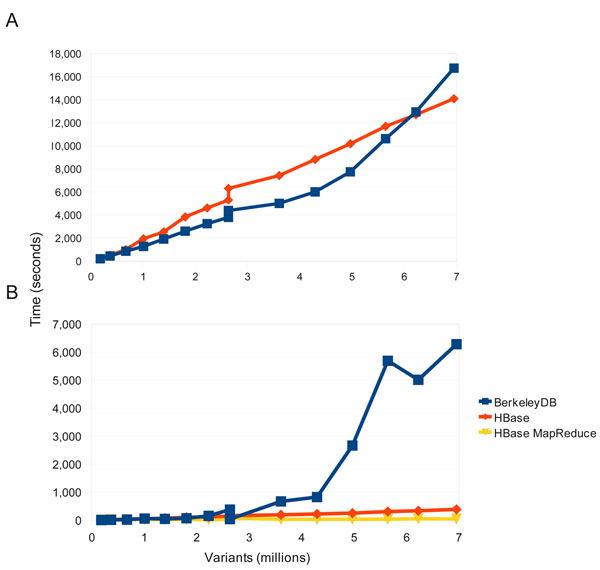
**Load and query performance.** Comparisons of load and query times between the HBase and BerkeleyDB backend. (a) Load times for the “1102 GBM” tumor/normal genomes where compared between HBase and BerkeleyDB. Both used a single-threaded approach to better compare relative performance. Both perform similarly but over time the load times for BerkeleyDB increase faster than with HBase. (b) Comparison of querying the 1102 genome database between BerkeleyDB, HBase single threaded, and HBase using MapReduce. Beyond 3M variants BerkeleyDB query times increase dramatically while both query types for HBase perform linearly, with MapReduce consistently exhibiting the best performance.

Figure 2 shows a comparison between the BerkeleyDB and HBase backends for both variant load and variant export functions. BerkeleyDB was chosen as the original backend for multiple reasons: it did not require a database daemon to run, it provided a key-value store similar to the distributed key-value NoSQL stores we intended to move towards, it had a well-designed API, and it was known to be widely used and robust. In the load tests both BerkeleyDB and HBase performed comparably (Figure 2a), with both tests using a single thread with a similar client API (rather than a MapReduce loader which would be possible only with HBase). BerkeleyDB is slightly faster until about 6 million (6M) variants are loaded but HBase is faster after that point and eventually takes about 15 minutes less to load all 7M variants in this test. The test for variant export had a significantly different outcome (Figure 2b). Both MapReduce and standard single-thread API retrieval of variants from HBase were extremely efficient, with the MapReduce exporter completing in 45 seconds and the single-thread in 386 seconds. This is in sharp contrast with the BerkeleyDB backend which took 6,281 seconds to export the 7M variant records. When the BerkeleyDB database reached approximately 3.5M variants the run time to export ballooned quickly. This was likely due to memory limitations on the single node serving the BerkeleyDB, resulted in greatly degraded query performance once the index could no longer fit in memory. In contrast, the HBase cluster nodes were each responsible for storing and querying only a fraction of the data and this data sharding resulting in much more robust query performance. Rather than making a statement about the inherent merits of the database systems themselves, this results underscores the point that the distributed HBase/Hadoop implementation (in this case spread across 6 nodes) has clear scalability advantages compared to daemons or processes like BerkeleyDB that are limited to a single server.

### Software availability

The SeqWare Query Engine is a sub-project of the SeqWare toolset for next generation sequence analysis. SeqWare includes a LIMS, pipeline based on Pegasus [26], and metadata schema in addition to the query engine. Like the rest of the SeqWare project, SeqWare Query Engine is fully open source and is distributed under the terms of the GNU General Public License v3 (http://www.gnu.org/licenses/licenses.html). The software can be downloaded from version control on the project**’**s SourceForge.net site (http://seqware.sourceforge.net). Visitors to the site can also post questions on the mailing list, view documentation on wiki pages, and download a pre-configured SeqWare Pipeline and Query Engine as a virtual machine, suitable for running in a wide array of environments. The present authors are very interested in working with other developers on this project and welcome any contributions.

## Discussion

Recent innovations from search-oriented companies such as Google, Yahoo, Facebook, and LinkedIn provide compelling technologies that could potentially enable computation on petascale sequence data. Projects such as Hadoop and HBase, open source implementations of Google**’**s MapReduce framework [27] and BigTable database [28] respectively, can be converted to powerful frameworks for analyzing next generation sequencing data. For example, MapReduce is based on a functional programming style where the basic methods available to perform analysis are a map phase, where data is transformed from one form to another, and a reduce phase, where data is sorted and condensed. This fits well to fundamental sequence analysis computations such as alignment and variant calling. Already there are versions of analysis algorithms such as Crossbow (alignment, [29]) and GATK (variant calling and other tools, [30]) that make use of the MapReduce paradigm. The approach is not fundamentally different from other functional programming languages that have come before, but in this case the approach is combined in Hadoop with both a distributed filesystem (HDFS) and tightly coupled execution engine. These tools, in turn, form the basis of the HBase database system. Unlike traditional grid technologies—for example a sun grid engine computation cluster—the Hadoop environment automatically partitions large data files across the underlying cluster, and computations can then be distributed across the individual data pieces without requiring the analysis program to know where the data resides, or manage such information. The HBase database system—one of the most mature of the NoSQL database projects—takes advantage of this sharding feature and allows a database to be broken into distinct pieces across the underlying computer cluster. This enables the creation of much larger databases than can be supported in traditional relational implementations which are constrained to run on a single database server, up to the scale of billions of rows (e.g. bases in a genome) and millions of columns (e.g. individual genomes). This is considerably larger than most database systems typically support, but is the correct scale needed to represent heavily annotated whole genome sequence data for future large scale biomedical research studies and clinical deployment to the broadest patient populations.

## Conclusions

Here we have introduced the open source SeqWare Query Engine that uses an HBase backend and the MapReduce/Hadoop infrastructure to provide robust databasing of sequence data types for the SeqWare project. The results show the scaling benefits that result from these highly distributable technologies even when creating a database of genomic variants for just 14 human genome datasets. The basic database functions, such as importing and exporting, are one to two orders of magnitude faster with HBase compared to BerkelyDB. Moreover, the highly nonlinear improvement in scaling is readily demonstrated at the critical point where the standard database server becomes saturated, whereas the HBase server maintains a proper distributed load as the data burden is increased. This fully cloud-oriented database framework is ideal for the creation of whole genome sequence/variant databases. It is capable of supporting large scale genome sequencing research projects involving hundreds to thousands of genomes as well as future large scale clinical deployments utilizing advanced sequencer technology that will soon involve tens to hundreds of thousands of genomes.

## List of abbreviations

ACID: atomicity, consistency, isolation, and durability; API: Application Programming Interface; BED: Browser Extensible Data (encodes variants); ChIP-Seq: Chromatin Immunoprecipitation sequencing; CSS: Cascading Style Sheets; GATK: Genome Analysis Toolkit; GBM: Glioblastoma Multiforme; GFF: General Feature Format (encodes genomic features); HDFS: Hadoop Distributed File System; IGV: Integrative Genomics Viewer; indel: small (typically <10bp) insertion or deletion; REST: Representational State Transfer; RNA-Seq: RNA sequencing; SNP: Single Nucleotide Polymorphism; SNV: Single Nucleotide Variant; SQL: Structured Query Language; SV: Structural Variants; TCGA: the Cancer Genome Atlas; URL: Uniform Resource Locator; VCF: Variant Call Format; XML: Extensible Markup Language; XSLT: XSL Transformations

## Competing interests

The authors have declared no competing interests.

## Authors’ contributions

BDO designed and implemented the SeqWare Query Engine. BM provided guidance on project goals, applications, selection of annotation databases, and choice of analysis algorithms used. SFN is the principal investigator for the U87MG genome sequencing project which supported the development of the SeqWare project including the query engine.

## References

[B1] Snyder M Du J Gerstein M Personal genome sequencing: current approaches and challenges Genes & development 2010 24 5 423 10.1101/gad.1864110 20194435 PMC2827837

[B2] Lander E Linton L Birren B Nusbaum C Zody M Baldwin J Devon K Dewar K Doyle M FitzHugh W Initial sequencing and analysis of the human genome Nature 2001 409 6822 860 921 10.1038/35057062 11237011

[B3] Levy S Sutton G Ng P Feuk L Halpern A Walenz B Axelrod N Huang J Kirkness E Denisov G The diploid genome sequence of an individual human PLoS Biol 2007 5 10 e254 10.1371/journal.pbio.0050254 17803354 PMC1964779

[B4] Wheeler D Srinivasan M Egholm M Shen Y Chen L McGuire A He W Chen Y Makhijani V Roth G The complete genome of an individual by massively parallel DNA sequencing Nature 2008 452 7189 872 876 10.1038/nature06884 18421352

[B5] Pushkarev D Neff N Quake S Single-molecule sequencing of an individual human genome Nature biotechnology 2009 27 9 847 850 10.1038/nbt.1561 19668243 PMC4117198

[B6] Wang J Wang W Li R Li Y Tian G Goodman L Fan W Zhang J Li J Zhang J The diploid genome sequence of an Asian individual Nature 2008 456 7218 60 65 10.1038/nature07484 18987735 PMC2716080

[B7] Bentley D Balasubramanian S Swerdlow H Smith G Milton J Brown C Hall K Evers D Barnes C Bignell H Accurate whole human genome sequencing using reversible terminator chemistry Nature 2008 456 7218 53 59 10.1038/nature07517 18987734 PMC2581791

[B8] McKernan K Peckham H Costa G McLaughlin S Fu Y Tsung E Clouser C Duncan C Ichikawa J Lee C Sequence and structural variation in a human genome uncovered by short-read, massively parallel ligation sequencing using two-base encoding Genome research 2009 19 9 1527 10.1101/gr.091868.109 19546169 PMC2752135

[B9] Ahn S Kim T Lee S Kim D Ghang H Kim D Kim B Kim S Kim W Kim C The first Korean genome sequence and analysis: full genome sequencing for a socio-ethnic group Genome research 2009 19 9 1622 10.1101/gr.092197.109 19470904 PMC2752128

[B10] Kim J Ju Y Park H Kim S Lee S Yi J Mudge J Miller N Hong D Bell C A highly annotated whole-genome sequence of a Korean individual Nature 2009 460 7258 1011 1015 19587683 10.1038/nature08211 PMC2860965

[B11] Drmanac R Sparks A Callow M Halpern A Burns N Kermani B Carnevali P Nazarenko I Nilsen G Yeung G Human genome sequencing using unchained base reads on self-assembling DNA nanoarrays Science 2010 327 5961 78 10.1126/science.1181498 19892942

[B12] Ley T Mardis E Ding L Fulton B McLellan M Chen K Dooling D Dunford-Shore B McGrath S Hickenbotham M DNA sequencing of a cytogenetically normal acute myeloid leukaemia genome Nature 2008 456 7218 66 72 10.1038/nature07485 18987736 PMC2603574

[B13] Mardis E Ding L Dooling D Larson D McLellan M Chen K Koboldt D Fulton R Delehaunty K McGrath S Recurring mutations found by sequencing an acute myeloid leukemia genome New England Journal of Medicine 2009 361 11 1058 10.1056/NEJMoa0903840 19657110 PMC3201812

[B14] Pleasance E Stephens P O’Meara S McBride D Meynert A Jones D Lin M Beare D Lau K Greenman C A small-cell lung cancer genome with complex signatures of tobacco exposure Nature 2010 463 184 190 10.1038/nature08629 20016488 PMC2880489

[B15] Pleasance E Cheetham R Stephens P McBride D Humphray S Greenman C Varela I Lin M Ordóñez G Bignell G A comprehensive catalogue of somatic mutations from a human cancer genome Nature 2010 463 191 196 10.1038/nature08658 20016485 PMC3145108

[B16] Clark M Homer N O’Connor B Chen Z Eskin A Lee H Merriman B Nelson S U87MG decoded: the genomic sequence of a cytogenetically aberrant human cancer cell line PLoS Genet 2010 6 e1000832 10.1371/journal.pgen.1000832 20126413 PMC2813426

[B17] Rhead B Karolchik D Kuhn R Hinrichs A Zweig A Fujita P Diekhans M Smith K Rosenbloom K Raney B The UCSC genome browser database: update 2010 Nucleic Acids Res 2010 38 Database issue D613 D619 10.1093/nar/gkp939 19906737 PMC2808870

[B18] Mungall C Emmert D A Chado case study: an ontology-based modular schema for representing genome-associated biological information Bioinformatics 2007 23 13 i337 10.1093/bioinformatics/btm189 17646315

[B19] Hubbard T Aken B Beal K Ballester B Caccamo M Chen Y Clarke L Coates G Cunningham F Cutts T Ensembl 2007 Nucleic acids research 2006 10.1093/nar/gkl996 PMC1761443 17148474

[B20] Kent W Sugnet C Furey T Roskin K Pringle T Zahler A The human genome browser at UCSC Genome research 2002 12 6 996 12045153 10.1101/gr.229102 PMC186604

[B21] Stein L Mungall C Shu S Caudy M Mangone M Day A Nickerson E Stajich J Harris T Arva A The generic genome browser: a building block for a model organism system database Genome research 2002 12 10 1599 10.1101/gr.403602 12368253 PMC187535

[B22] Karolchik D Hinrichs A Furey T Roskin K Sugnet C Haussler D Kent W The UCSC Table Browser data retrieval tool Nucleic acids research 2004 32 Database Issue D493 10.1093/nar/gkh103 14681465 PMC308837

[B23] Giardine B Riemer C Hardison R Burhans R Elnitski L Shah P Zhang Y Blankenberg D Albert I Taylor J Galaxy: a platform for interactive large-scale genome analysis Genome research 2005 15 10 1451 10.1101/gr.4086505 16169926 PMC1240089

[B24] Fielding R Architectural Styles and the Design of Network-based Software Architectures PhD thesis 2000 University of California

[B25] Li H Handsaker B Wysoker A Fennell T Ruan J Homer N Marth G Abecasis G Durbin R The sequence alignment/map format and SAMtools Bioinformatics 2009 25 16 2078 10.1093/bioinformatics/btp352 19505943 PMC2723002

[B26] Deelman E Singh G Su M Blythe J Gil Y Kesselman C Mehta G Vahi K Berriman G Good J Pegasus: A framework for mapping complex scientific workflows onto distributed systems Scientific Programming 2005 13 3 219 237

[B27] Dean J Ghemawat S MapReduce: Simplified data processing on large clusters Communications of the ACM 2008 51 107 113 10.1145/1327452.1327492

[B28] Chang F Dean J Ghemawat S Hsieh W Wallach D Burrows M Chandra T Fikes A Gruber R Bigtable: A distributed storage system for structured data ACM Transactions on Computer Systems (TOCS) 2008 26 2 4 10.1145/1365815.1365816

[B29] Langmead B Schatz M Lin J Pop M Salzberg S Searching for SNPs with cloud computing Genome Biology 2009 10 11 R134 10.1186/gb-2009-10-11-r134 19930550 PMC3091327

[B30] McKenna A Hanna M Banks E Sivachenko A Cibulskis K Kernytsky A Garimella K Altshuler D Gabriel S Daly M The Genome Analysis Toolkit: A MapReduce framework for analyzing next-generation DNA sequencing data Genome Research 2010 20644199 10.1101/gr.107524.110 PMC2928508

